# Weight-bearing recommendations after first metatarsophalangeal joint arthrodesis fixation: a biomechanical comparison

**DOI:** 10.1186/s13018-017-0525-z

**Published:** 2017-02-06

**Authors:** Bradley Campbell, Patrick Schimoler, Sudhir Belagaje, M. C. Miller, S. F. Conti

**Affiliations:** 1 Department of Mechanical Engineering and Material Science, University of Pittsburgh, 3700 O’Hara Street, Pittsburgh, PA 15261 USA; 2 Department of Orthopaedic Surgery, Orthopaedic Biomechanics Laboratory, Allegheny General Hospital, 320 E. North Ave, Pittsburgh, PA 15212 USA; 3 Department of Bioengineering, University of Pittsburgh, 3700 O’Hara Street, Pittsburgh, PA 15261 USA; 4 Orthopaedic Practices, Pittsburgh, PA 15261 USA

**Keywords:** Foot, Metatarsal, Hallux rigidus, Weight-bearing, Fusion

## Abstract

**Background:**

This study sought to determine whether several metatarsophalangeal (MTP) fusion techniques require complete immobilization or if some level of weight-bearing could be recommended after surgery. A comparison of synthetic composite to actual bone was included in order to examine the validity of the testing conditions.

**Methods:**

Four MTP fusion modalities were tested in synthetic composite bone models: unlocked plating, locked plating, crossed lag screws, and an unlocked plate with a single lag screw. Stiffness was calculated and then used to find the two most rigid constructs; the load to failure was recorded. Stiffness and load to failure testing for the two more rigid constructs in paired cadaveric bones were followed.

**Results:**

The unlocked plate plus screw and crossed screw constructs were stiffest (*p* < 0.008). Loads to failure of the unlocked plate plus screw and crossed screws in synthetic bone were 131 and 101 N, respectively and in cadaveric bone were 154 and 94 N, respectively, which are less than the estimated 25% body weight required at the MTP joint. The plate plus screws were statistically more stiff than crossed screws (*p* = 0.008), but there was no statistical difference between synthetic and cadaveric bone in load to failure (*p* = 0.296).

**Conclusions:**

The plate plus screw offered the greatest stiffness; the failure test showed that no construct could withstand weight-bearing as tolerated; and, synthetic composite models of the MTP joint did not provide the consistent results in stiffness and failure.

## Background

Late stage hallux valgus and hallux rigidus are commonly treated in osteoarthritic patients with arthrodesis using mechanical hardware to stabilize the joint [1–4]. Post-surgery protocol includes either non-weight-bearing or weight-bearing in a postoperative shoe to avoid high pressure under the metatarsophalangeal (MTP) joint [5–7]. Patients are expected to mitigate the impact of any gait loading on the great toe.

Studies have suggested immediate weight-bearing may be acceptable and is preferable [8, 9], though the extent of allowable weight-bearing is unknown. The subhallucal load can be 25% of body weight at toe off [10] during normal gait, and, although touch-down weight-bearing and the use of assistive devices could reduce this demand, a large upper limit on the allowable load would ensure greater confidence in the outcome. A minimum estimate of the hallux load for a 784 N patient, the average body weight for a male [11], would thus lead to 196 N of load beneath the first MTP joint, which any MTP fusion construct would have to support.

The overall goal of the current research was to estimate allowable post-operative weight-bearing with four MTP fusion techniques with the purpose of providing sound reasoning for post-operative weight-bearing recommendations. The comparison of four different fixation modalities complicated paired specimen testing conditions. Literature has shown composite bones, in particular, fourth generation synthetic composite models, and human bones can behave similarly under cyclic and failure loading if the tests are carefully designed [12–14]. Even so, in that cadaveric bone testing is the most accepted method and that even the use of paired specimens cannot ensure uniform testing conditions for an assessment of four different techniques, an experimental design utilizing comparisons in synthetic composite bone with a parallel comparison test in cadaveric bone using two of the techniques was chosen. Thus, the experimental design included four cases tested with synthetic composites and then a comparison with the two most promising techniques tested in cadaveric bone. The testing encompassed stiffness measurement and load to failure testing. That is, malunion or non-unions may be related to cyclic failure of the components occurring in non-compliant patients from premature loading [15, 16], so considerations of cyclic loading are important. For ultimate failure of the fusion, to consider non-union as the failure in this in vitro test, the failure criterion was taken to be 2 mm of relative movement between any part of the articulation at the joint. The failure criterion of 2 mm is supported by similar criteria in the literature [3, 17, 18].

The basic research questions for the study concerned the problems of weight-bearing. First, we sought to determine which common fixation was the stiffest, offering the most resistance in repetitive loading to the opening of the fused joint, and whether the stiffest constructs could tolerate post-operative weight-bearing. Additionally, the work sought to contribute to the understanding of test methodology by considering whether synthetic composite bones provide an adequate means for comparison of MTP fixation techniques. The underlying null hypotheses were that no differences existed between MTP fixation techniques or between testing in cadaveric or synthetic composite bone.

## Methods

### Specimen preparation

Four commonly used hardware configurations were compared: (i) an unlocked plate held by four screws, (ii) a locked plate held by four screws and two locking washers, (iii) an unlocked plate held by four screws combined with a single lag screw, and (iv) a pair of lag screws for crossed screw fixation across the joint. First metatarsal and proximal phalanx synthetic bones were obtained (models 3422 and 3423, Pacific Resources, Vashon WA) and prepared for metatarsal-phalangeal fusion. Prior to fixation, the outer layer of the synthetic bone material was removed from the articular surface.

A board certified, fellowship-trained orthopaedic surgeon reamed the surfaces, determined appropriately sized hardware, and completed the fixation. For cases of plate fixation, the metatarsal and proximal phalanx were templated for hole placement and screw length. The plate was implanted and, in the case of the locked plate, a locking screw construct was placed in the first and fourth holes of the plate. For lag screw fixation, a Kirschner wire was obliquely inserted into the proximal end of the lateral phalanx to guide the cannulated lag screw through the joint line into the distal medial metatarsal. In the case of plate fixation with a single crossed screw, the screw was added after plate fixation.

**Fig. 1:**
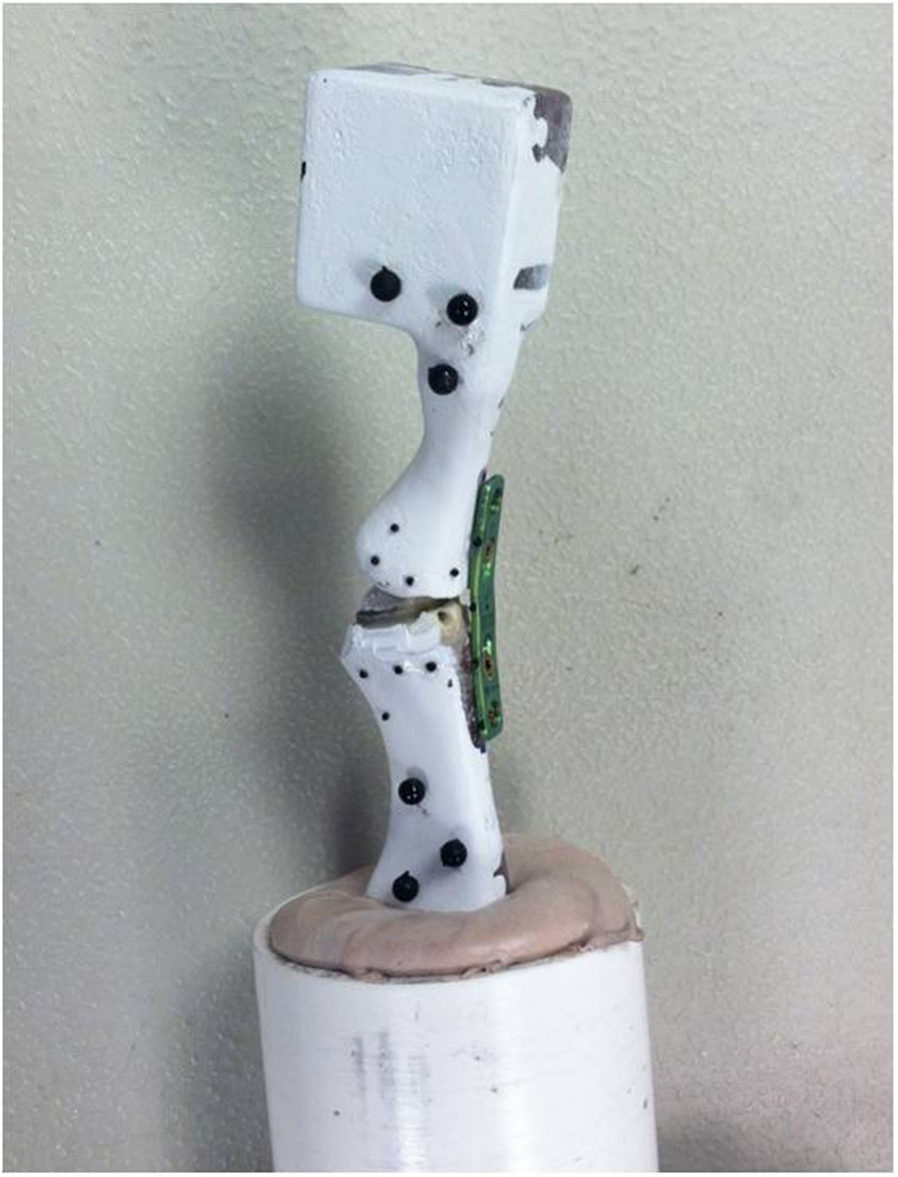
An image of the marked, painted synthetic composite bones fixed in 2-inch-diameter polyvinyl chloride piping and filled with polymer resin and organic peroxide (Bondo, 3M, St. Paul, MN). The constructs were labeled with markers for the camera tracking. The cadaveric specimens were similarly potted and marked for camera tracking

After painting all specimens white to assist in subsequent optical tracking, the proximal end of the metatarsal was placed into a 2-inch-diameter polyvinyl chloride pipe filled with polymer resin and organic peroxide (Bondo, 3M, St. Paul, MN). Upon hardening, the metatarsal was firmly held in the polyvinyl cylinder. For measurement of the opening of the joint space between the phalanx and metatarsal, black spherical markers (1.5 mm in diameter) were fixed at the joint line of the construct with a cyanoacrylate-based adhesive (Prism, Loctite, Germany). Four 3-mm-diameter spheres were fixed laterally to both the metatarsal and phalanx to simplify gross measurement of the movement of each bone, as needed for tracking each body (Fig. 1).

**Fig. 2:**
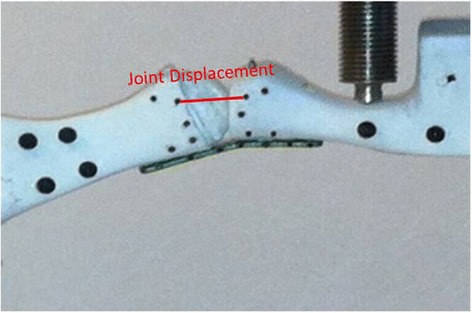
An image of the marked, painted Sawbones composite bones being loaded. The constructs were labeled with markers for the camera tracking. The joint displacement was determined by measuring the distance between the identified markers throughout the testing trial

Cadaveric specimens to test the validity of the results from the composite bones were similarly prepared. Five pair of frozen feet were allowed to completely thaw and prepared by the same fellowship-trained orthopaedic surgeon (average specimen age 71 ± 15 years; 3 males, 2 females) All specimens were from ambulatory donors with neither foot or ankle pathology nor any disease that would affect bone quality, such as cancer or long-term complete immobilization. A dorsal medial incision was made to expose the metatarsophalangeal joint for fixation. As in the preparation of the synthetic bones, the lag screw fixation followed plate fixation in the unlocked plate plus screw case and Kirschner wires were used to guide both lag screws in the use of crossed screws. After implantation, the first and second rays of each cadaveric foot were excised from the whole foot. The intact hallux, the cuneiforms, phalanx, and metatarsal were removed together. The proximal ends of the metatarsal and the cuneiforms were placed into 2-inch-diameter polyvinyl chloride pipe and filled with polymer resin and organic peroxide (Bondo, 3M, St. Paul, MN). Black spherical markers for tracking of joint opening and body movement were placed laterally along the joint line (Fig. 2).

### Biomechanical testing

Each construct was placed in an axial load frame (Bionix 858, MTS, Eden Prairie, MN) with the metatarsal positioned 15° from the horizontal to simulate the position of the metatarsal with respect to the ground during upright standing. Based on the literature [3, 18, 19], the failure criterion was taken to be a 2-mm opening at anywhere along the joint line, i.e., the amount of movement at the fusion site leading to a non-union. Preliminary testing was performed to find the minimal load to cause 2-mm displacement (i.e., the non-union) at the joint in the four constructs. This preliminary test found that 40 N of dorsally directed load in the case of the unlocked plate led to 2 mm of joint opening on the plantar side of the phalanx. A 40 N load, directed plantar to dorsal, was then applied at 1 Hz to all specimens for 1000 cycles of loading, and the movement of the spherical markers was tracked optically (accuracy: 0.1 mm; Spica Technology Corporation, Kihei, Maui, HI) at 10 Hz. The data acquisition of the load and position were synchronized by the simultaneous input of a step signal into each data set. The output from the tests included the three-dimensional position of the markers, the force applied and the time information related to each test.

Based on the results of composite bone testing, the two stiffest constructs were applied to paired cadaveric feet, and the two least stiff were set aside for use in an unrelated experiment. The cadaveric specimens were tested with the joint opening protocol. Finally, each of the constructs was mounted in the uniaxial load frame and linearly increasing load was applied until the specimen failed, e.g., screw cutout or pullout occurred. The largest load was recorded for each specimen.

### Data analysis

The joint opening during cyclic loading was calculated using the movement occurring between the phalanx and the metatarsal at the joint line. The opening at the plantar edge of the joint line that occurred with the maximum load of each cycle in the group was always the largest and was extracted from the data for each case. The stiffness was determined by dividing the load difference between the peak and minimum load values of each cycle by the displacement occurring between the peak and minimum with the average taken over 30 s intervals at the beginning, middle, and end of the test. All calculations and analysis were performed in Matlab.

### Statistical analysis

A Kruskal-Wallis test was performed to examine the relationship of stiffness (the dependent variable) with fixation type (the independent variable) using IBM SPSS, Version 23 (Armonk, NY). If the initial comparison showed significance, individual Mann-Whitney comparisons with Bonferroni correction were used as post hoc tests to compare each pairing of fixation types. Failure loads were compared with *t* tests. A *p* value of less than 0.05 in comparison was considered significant.

## Results

The use of synthetic composite bones was necessary to maintain the same conditions for each of four fixation methods. The subsequent tests in paired cadaveric bone of two of the four fixations were performed to estimate whether the composite bone results were replicated in actual specimens. After the cyclic loading of the synthetic composite specimens, the two stiffest constructs proved to be the plate plus screw and the use of two crossed screws. These fixation techniques were tested to failure in the paired cadaveric specimens and both the two stiffest synthetic composite bone paired cadaveric specimens were loaded to failure.

The tests of the composite bones with 40 N of loading showed (1) that the largest displacement occurred in the case of the unlocked plate, and (2) that the two stiffest constructs were the dorsal plate with the single screw across the joint and the crossed screws (Table 1). Over all four constructs, the dependent variable of stiffness was significant in the Kruskal-Wallis analysis (*p* = 0.0001). Individual multiple comparisons tests on the pairs of data sets showed differences between all pairs of fixation types (*p* < 0.008 for all) except for the locked to unlocked plate fixation comparison, which showed no significance, *p* = 0.463.

**Table 1:** Stiffness and failure data from both synthetic bone and cadaveric bone tests

		Cyclic loading	Failure loading
Synthetic		Stiffness (N/mm)	Maximum allowable load (N)
	Unlocked	15.6 (±1.7)	N/A
	Locked	18.1 (±2.3)	N/A
	Crossed screws	94.7 (±12.5)	101.0 (±17.8)
	Plate and lag screw	373.4 (±76.3)	130.9 (±19.4)
Cadaveric		Stiffness (N/mm)	Maximum allowable load (N)
	Crossed screws	152.4 (±14.2)	93.9 (±14.4)
	Plate and lag screw	122.1 (±5.9)	154.1 (±40.7)

This table shows average stiffness, average failure loads, and corresponding standard error for all four constructs tested. The unlocked plate plus screw construct was stiffer than the other constructs for those assembled with the composite bones. Using cadaveric bones did not support that finding with the crossed screws construct having a higher measured stiffness than the unlocked plate plus screw

Comparing the stiffness measured at the beginning of the test with the stiffness at the end of the test, stiffness was unchanged over 1000 cycles of loading in either synthetic composite or cadaveric bone. The stiffnesses in cadaveric specimens were not within 20% of the stiffnesses in synthetic bone (Table 1). The dependent variable of stiffness was significant in the Kruskal-Wallis analysis. (*p* = 0.001) The stiffnesses of the plate plus screw and crossed screws in cadaveric bone were not statistically different from each other (*p* = 0.08). The plate plus screw in synthetic bone was stiffer than the crossed screws, but the opposite was true in cadaveric bone. Individual Mann-Whitney tests on the pairs of data sets showed differences between all pairs of fixation types (*p* = 0.012 for all) except for the locked to unlocked plate fixation comparison, which showed no significance, *p* = 0.403.

In load to failure testing, there was no statistical difference between the cases in either synthetic composite or cadaveric bone (*p* = 0.296). Moreover, neither the synthetic composite nor the cadaveric bones could reach weight-bearing load levels without catastrophic failure (Table 1). No construct could withstand 196 N, the estimated load on the phalanx for an average male.

## Discussion

Successful surgery can lead to good foot function without compounding stresses in other regions of the foot [20], but post-operative non-compliance and inadequate fixation can lead to complications such as non-union [21–23]. Nonetheless, satisfaction with arthrodesis has been reported to be over 80% [24–26] and fusion rates have been reported at up to 95% [8, 24, 27–30]. The ability to bear weight earlier could reduce complications due to patient non-compliance.

Orthopaedic surgeons have had little science to establish a weight-bearing protocol following first MTP fusion even though returning to early weight-bearing has many advantages to the recovering patient [2, 28]. In this study, the possibility of weight-bearing after arthrodesis of the 1st metatarsophalangeal joint was considered by measuring the actual load to failure. In order to have a consistent test platform for four constructs, synthetic composite bones were tested first. Then, to examine the validity of the synthetic composite tests, the two stiffest constructs from the initial test were tested in paired cadaveric bone.

That the plate plus lag screw was stiffest construct (*p* = 0.0001) should be expected because the plate plus screw fixation forms a composite beam structure when implanted, with bone between the plate and screw. This structure is much like an I-beam, where the flanges forming the top and bottom of the beam are stiffer in bending than the web between them. The location of the crossed screws in the central region of the bones meant that the distance from the hardware to the measurement site was smaller for the screws than for the single plates. Although the use of the joint opening as the measure of displacement may have favored the use of lag screws over the single, anteriorly mounted plates, in each fixation case, the measurements were taken at the point where maximal displacement would occur. In effect, the single plates only resisted bending due to the plate and rotation of the bones about the hardware location occurred, as it would in vivo. A greater distance to the plantar edge of the joint meant, that for the same amount of rotation, the single plates would have greater opening. Assuming that the lag screws are on the midline and the plate is on the dorsal surface, the single plates are approximately twice as far from the point of measurement as the lag screws. Then, despite the seeming advantage of bone sandwiched between a plate and screw, the cadaveric tests of comparison found that the crossed-screw fixation was stiffer than the plate plus screw, although without statistical significance. This difference between the cadaveric and the composite bone could be due the extent to which lag screw fixation can join the MTP joint: if the lag screw is the limiting factor, the advantage of location for the crossed screws could dominate the positive effect of the hardware separation distance of the plate and screw construct.

The failure loads of plate plus screw and crossed screws were smaller and considerably less than the estimated load arising in weight-bearing gait. Simply stated, none of the constructs could sustain the weight-bearing load of 25% body weight of an average male, as evidenced by both the failure loads in synthetic composite and cadaveric bone. Thus, none of the constructs would suffice for weight-bearing similar to normal gait.

The results from the synthetic composite and cadaveric bones testing compared favorably in failure loads. The constructs failed at similar loads and showed that the plate plus screw is stronger than crossed screws in both cases. The stiffness result in synthetic composite was not replicated in cadaveric bone, however. Not only were the calculated values in synthetic composite and cadaveric bone dissimilar, but the crossed screws were stiffer than a plate plus screw in the cadaveric bone, contrary to the synthetic composite result. Much like the explanation for the differences in stiffness between the MTP fusion techniques, one possible explanation would be a difference in the lag screw fixation in the two materials. While Heiner [14] found general agreement with real bone in stiffness of the whole structure of a synthetic bone model, Dunning found a disagreement between synthetic bone and actual bone in impact testing [31]. The disagreement of the current tests in stiffnesses but the agreement in failure loads could have been caused by the lack of homogeneity in the cadaveric bone taken from older donors. Synthetic specimens represent high quality bone and do not have the flaws, inconsistencies, and morphological differences found in actual cadaveric specimens. While consistency of the synthetic materials allows the concurrent study of multiple constructs, extreme care must be used in the choice of test protocols.

The use of synthetic composite bone could have been the major limitation of this work. The comparison to cadaveric bone, however, offered a means to assess the results and offer a separate measurement. Another potential limitation was that the sample size of five in each group of synthetic bone could potentially limit the study’s scope, but stiffness results proved to be statistically significant. This significance may have been the direct result of the more uniform specimens provided by the synthetic composite bone and then by the use of paired cadaveric specimens. A statistically significant outcome provides more confidence in the result. The lack of any real fusion also forms a limitation of the current study. As an in vitro study, only the period of time immediately after surgery could be tested. The strength of the in vivo construct will be time dependent, in that as fusion progresses over 6 weeks, and there is additional strength added to the construct by the progressive bone fusion. Finally, as a limitation, the estimate of the maximum required load borne by the hallux was derived from values in other research. However, the predictions of Gefen et al. [32] indicate that the 196 N load for a 784 N body weight have intuitive appeal.

## Conclusions

While the current results clearly advise against full weight-bearing at time zero, the stiffness and strength of a plate plus screw and of the crossed screws might tolerate protected weight-bearing if some means to ensure patient compliance were available. Single dorsal plates, whether locked or unlocked, would not be advisable for any form of weight-bearing. The orthopaedic literature has previously described the stiffness and strength of various first metatarsophalangeal constructs used in fusion. While this literature often suggests that the strongest construct would also have the best rate of fusion, to our knowledge, the relationship of stiffness and strength to fusion success has never been proven.
